# Camouflaging in autism: A cause or a consequence of mental health difficulties?

**DOI:** 10.1177/13623613251347104

**Published:** 2025-06-28

**Authors:** Wikke J van der Putten, Audrey JJ Mol, Tulsi A Radhoe, Carolien Torenvliet, Joost A Agelink van Rentergem, Annabeth P Groenman, Hilde M Geurts

**Affiliations:** 1 Leo Kannerhuis, Autism Outpatient Clinic(Youz/Parnassia Group), Amsterdam, The Netherlands; 2 University of Amsterdam, The Netherlands

**Keywords:** autism, camouflaging, mental health difficulties

## Abstract

Camouflaging ([un]consciously hiding one’s autism traits) is hypothesized to be an underlying mechanism explaining elevated levels of mental health difficulties in autistic adults. As previous studies investigating this relationship were all cross-sectional, the direction of this association remains unclear. Therefore, we investigated whether (1) camouflaging predicts a change in mental health difficulties and (2) mental health difficulties predict a change in camouflaging. For this, 332 autistic adults aged 30 to 84 years (157 women) filled in the Dutch Camouflaging Autistic Traits Questionnaire, Symptom Checklist–90 Revised, and Autism Spectrum Quotient at two timepoints with an average time interval of 2 years. Preregistered multilevel analyses showed a negative interaction between initial camouflaging and time, indicating that people with higher initial levels of camouflaging showed a decrease in mental health difficulties, while for lower initial levels of camouflaging there was an increase. However, this effect was small. Initial mental health difficulties did not predict changes in camouflaging behavior, but findings were inconsistent across total camouflaging and assimilation. Thus, we did not find strong evidence for camouflaging to be associated with development of mental health difficulties. However, future research is needed before we can draw strong conclusions about directionality and causality.

**Plain Language Summary:**

When autistic people use strategies to hide their autism traits, we refer to this as camouflaging. It has been thought that camouflaging could be a reason why autistic people develop mental health difficulties more often than non-autistic people. Research has shown that, in general, people who report more camouflaging behavior also report more mental health difficulties. However, we do not know whether camouflaging can be a reason for people to develop mental health difficulties or whether mental health difficulties may explain why autistic people use camouflaging strategies. Therefore, in this study we investigated whether (1) camouflaging is a predictor for a change in mental health difficulties and (2) mental health difficulties are a predictor for a change in camouflaging. For this study, 332 autistic adults aged 30 to 84 years (157 women) filled in questionnaires about camouflaging, mental health difficulties, and autism traits at two moments with an average of 2 years between measurements. We found that people with a higher level of initial camouflaging showed a decrease in mental health difficulties, while for people with lower levels of initial camouflaging behavior there was an increase in mental health difficulties. However, this effect was small. Initial mental health difficulties did not seem to predict a change in camouflaging behavior. Thus, we did not find evidence that camouflaging was followed by an increase in mental health difficulties. Therefore, future research is needed before we can draw strong conclusions about what comes first and what causes what, camouflaging or mental health.

## Introduction

It has been established that autistic^1^ adults experience more co-occurring mental health conditions compared with non-autistic adults (Hossain et al., 2020), which can greatly affect their quality of life (Mason et al., 2018). Investigating mechanisms through which autistic people develop mental health difficulties is an important step toward understanding why they experience these high levels of mental health conditions and toward improving mental health care (Mandy, 2022). One potential factor that has received increased attention in the last years is camouflaging,^2^ which refers to using conscious or unconscious strategies to hide or minimize the visibility of someone’s autism traits to pass as non-autistic (Hull et al., 2017; Libsack et al., 2021). Camouflaging may consist of imitating behaviors used by peers, making scripts for social situations, finding alternative strategies for making eye contact, or taking on a whole different persona in certain situations (Hull et al., 2017; Libsack et al., 2021; Livingston et al., 2019). While it has been shown that camouflaging is associated with various mental health difficulties, such as depression, anxiety, and suicidality, it remains unclear whether camouflaging causes these mental health difficulties (Cook et al., 2021). In this study, we investigate the association between camouflaging and mental health difficulties over time to make a step toward understanding the mechanism(s) underlying this relationship.

Autistic people camouflage for different reasons, such as getting by at work, maintaining a job, finishing education, or to make friends (Cage & Troxell-Whitman, 2019; Hull et al., 2017; Livingston et al., 2019). Also, autistic people report that they camouflage because of the pressure that they feel to behave according to the expectations and social norms of a largely non-autistic society (Zhuang et al., 2023). By camouflaging, autistic people can avoid being rejected or bullied by others (Hull et al., 2017; Livingston et al., 2019). Furthermore, it has been hypothesized that camouflaging can be an automatic response to feeling stigmatized or marginalized (Pearson & Rose, 2021). While camouflaging can help some autistic people navigate through a non-autistic society, others have reported negative consequences of camouflaging (Cage & Troxell-Whitman, 2019; Libsack et al., 2021).

First of all, camouflaging could lead to missed or late autism diagnosis (Bargiela et al., 2016). When autistic people hide how they differ from others, they are less likely to be recognized as autistic and, therefore, may not receive the appropriate care when necessary (Lai & Baron-Cohen, 2015). Suppressing one’s authentic self can have a negative impact on someone’s self-esteem as it could increase the feeling that someone is not good enough the way they are (Bargiela et al., 2016; Hull et al., 2017). In addition, constantly monitoring how to behave requires a lot of energy and, therefore, camouflaging can be exhausting (Hull et al., 2017; Livingston et al., 2019). Finally, autistic people mentioned that when they camouflage, they become anxious about people discovering they are autistic (Chapman et al., 2022). A meta-ethnography summarized the lived experiences that autistic people had of the relationship between camouflaging and mental health difficulties (Field et al., 2024). Some autistic people reported unintended negative consequences of camouflaging, while others reported that the negative effects of camouflaging outweigh the stressful effects of not camouflaging in certain contexts. These descriptions of autistic adults could explain why there is a relation between camouflaging and mental health difficulties.

This hypothesized relationship has been investigated in cross-sectional studies (for a review, see Cook et al., 2021). While results vary across studies, the general trend is that people who report more camouflaging strategies also report more mental health difficulties, such as depression, anxiety, stress, suicidal thoughts, and lower general well-being. Of these, anxiety or depression has gained the most research traction (Cage & Troxell-Whitman, 2019; Hull et al., 2021; Lai et al., 2016). Furthermore, a few studies looked into types of camouflaging strategies one uses, as measured by the Camouflaging Autistic Traits Questionnaire (CAT-Q; Hull et al., 2019). In cross-sectional studies, medium-sized positive associations have been found between mental health difficulties with all subscales of the CAT-Q (more mental health difficulties are associated with more camouflaging behavior; for a meta-analysis, see Khudiakova et al., 2024). Especially the assimilation subscale (i.e. strategies that reflect trying to fit in with others in social situations) seemed most strongly related to various types of mental health difficulties (Cassidy et al., 2019; Hull et al., 2019). As the assimilation subscale has been shown to correlate most strongly to social anxiety, it has been hypothesized that assimilation strategies are used as a type of safety behavior against social anxiety. This suggests that assimilation strategies may be suitable to focus on in relation to mental health while exploring the influences of other subscales. Due to the cross-sectional study design of these studies, we cannot determine whether camouflaging predicts the development of mental health difficulties over time.

While the hypothesis that camouflaging is associated with the development of mental health difficulties over time is considered the most likely (Hull et al., 2017; Livingston et al., 2019), autistic people may also develop camouflaging strategies as a consequence of experiencing mental health difficulties. In a recent qualitative study, autistic teenagers reported that the association between camouflaging and mental health potentially goes both ways (Chapman et al., 2022). They reported that feeling anxious in social situations was a reason for them to camouflage, but that camouflaging also had negative consequences for their mental health. However, it has not yet been investigated with quantitative measures what comes first, camouflaging or the mental health difficulties. Knowing the direction of this association can have great implications for autistic people and clinical practice. When establishing whether there is a causal relationship between two factors, there are several criteria that have to be met (Albers & Kratochwill, 2010): (1) the cause occurs before the effect (temporal precedence), (2) the cause and effect are associated, and (3) there are no plausible alternative explanations. Based on the cross-sectional studies, it is plausible that at least an association between camouflaging and mental health will be found. It is important to first test whether temporal precedence can be observed. Subsequently, given the various known reasons to camouflage, one could test the different factors impacting this aforementioned association. A first step to investigate directionality (and eventually possibly causality) in the association between camouflaging and mental health is to use an observational longitudinal study design (Lervåg, 2019).

Therefore, the goal of this study is to use a longitudinal study design to investigate whether (1) initial level of camouflaging is a predictor for a change in mental health difficulties and (2) initial level of mental health difficulties is a predictor for a change in camouflaging. As mentioned before, we expect that assimilation is more closely related to anxiety, depression, and mental health difficulties compared with total camouflaging and total camouflaging and assimilation are more closely associated with depression and anxiety, compared with total mental health difficulties. Therefore, we investigate these hypotheses for camouflaging and mental health difficulties, but also specifically for assimilation, depression, and anxiety. Mental health difficulties are measured using the total score of the Symptom Checklist-90 Revised (SCL-90-R), which has been shown to measure general psychological distress, and the anxiety and depression subscales of the SCL-90-R are included to measure specifically anxiety and depression. With this study, we aim to take a next step toward disentangling the role that camouflaging plays in developing mental health difficulties. This knowledge could improve understanding of why autistic people experience more co-occurring mental health difficulties and provide insights for interventions to improve mental health in autistic people.

## Method

### Participants

All adults included in this study participated in a large longitudinal study investigating “Autism & Aging” (full procedure is described in Geurts et al., 2021). They met the following inclusion criteria: (1) a self-reported clinical autism diagnosis; (2) no intellectual disability or, when they had received an intelligence test in the past 5 years, a self-reported intelligence quotient above 70; (3) sufficient understanding of the Dutch language to fill in the questionnaires; and (4) between 30 and 90 years old at baseline (T1). Participants were recruited through mental health care institutions, patient advocacy organizations, social media, or via previous participation in studies of this research group.

On T1, 354 participants filled in all questionnaires for this study (see measures below). At follow-up, 12 participants did not want to participate anymore, two participants could not be reached, three participants passed away between measurements, three participants did not fill in all measurements necessary for this study at T2, and two participants were excluded because their diagnosis was disconfirmed since the first measurement. This resulted in a total sample of 332 adults who could be included for the study, see Table 1. The 22 people who dropped out did not significantly differ from the total sample at baseline in biological sex, age, AQ score, Dutch Camouflaging Autistic Traits Questionnaire (CAT-Q-NL) score, and SCL-90-R score, see Supplemental Table S1. Time between measurements was on average 2.0 years (*SD* = 0.3, range = 1.3–3.3).

**Table 1.** Characteristics of autistic adults included in this study.

	T1 (*N* = 332)	T2 (*N* = 332)
Biological Sex (m/f/o)^a,b^	174/ 157/ 1	-
Education^c^	101/ 136/ 94	-
	*M* (*SD*; range)	*M* (*SD*; range)
Age	52.4 (12.4; 30–84)	54.3 (12.3; 32–86)
Age of diagnosis	44.1 (13.1; 3–78)	-
Years since diagnosis	6.9 (5.0; 0–29)	-
AQ	34.7 (7.4; 10–48)	33.6 (7.7; 7–49)
CAT-Q-NL total	98.6 (26; 29–169)	97.4 (25.6; 32–170)
*Compensation*	31.4 (11.2; 9–63)	31.7 (11.2; 9–62)
*Masking*	31.9 (10.6; 8–56)	31.2 (10.1; 8–56)
*Assimilation*	35.3 (9.3; 9–54)	34.5(9.4; 9–54)
SCL-90-R total	169.6 (51.1; 93–397)	166.2 (54.7; 92–406)
*Anxiety*	18.2 (7.2; 10–48)	17.9 (7.5; 10–49)
*Depression*	33.0 (12.5; 16–77)	32.9 (13.5; 16–76)

m/f/o: male/female/other; AQ: Autism Spectrum Quotient; CAT-Q-NL: Dutch Camouflaging Autistic Traits Questionnaire; SCL-90-R: Symptom Checklist-90 Revised.

a Biological sex, education, and age of diagnosis are reported on T1 only.

b Next to biological sex, there was an open-ended question about gender. However, this did not result in a large enough group (*N* = 13) with a different gender identification compared with their biological sex to analyze separately.

c Education is classified using the Dutch Verhage scale ranging from 1 (less than 6 years of primary education) to 7 (university degree) (Verhage, 1964). The first five classes were merged to prevent empty cells and, therefore, include a range of education less than 6 years of primary education to practical higher education; the second cell refers to higher vocational education or pre-university education and the third to a university degree.

### Materials

#### Dutch Camouflaging Autistic Traits Questionnaire

The CAT-Q-NL (Hull et al., 2019; van der Putten et al., 2023a) is a self-report questionnaire that consists of 25 items describing different types of camouflaging. Participants indicated on a 7-point Likert-type scale whether they *strongly disagree* (1) to *strongly agree* (7) with each statement. The CAT-Q-NL measures three types of camouflaging: compensation (strategies used to actively compensate for difficulties in social situations), masking (strategies used to hide autistic characteristics or portray a non-autistic persona), and assimilation (strategies that reflect trying to fit in with others in social situations). The assimilation subscale (*N*_items_ = 8, range = 8–56) was used in this study together with the total CAT-Q-NL score (range = 25–175). The internal reliability of the CAT-Q-NL and its subscales ranges from sufficient to good (Cronbach’s α = 0.80 (assimilation) to 0.93 (total score); van der Putten et al., 2023a).

#### Symptom Checklist-90 Revised

The SCL-90-R (Arrindell & Ettema, 2005) is a multidimensional self-report questionnaire that measures the presence of current psychopathological symptoms and psychological distress, which we refer to as mental health difficulties. Items are rated on a 5-point Likert-type scale ranging from *not at all* (1) to *very much* (5). The SCL-90-R total score consists of the following subscales: Somatization, Obsessive Compulsive Disorder, Interpersonal Sensitivity, Depression, Anxiety, Hostility, Phobic Anxiety, Paranoid Ideation, and Psychoticism. For this study, we used total SCL-90-R score (*N*_items_ = 90, range = 90–450), the anxiety subscale (*N*_items_ = 10, range = 10–50), and depression subscale (*N*_items_ = 16, range = 16–80). Internal consistency of total SCL-90-R score, depression subscale, and anxiety subscale ranges from good to excellent (Cronbach’s α = 0.88 to 0.97; Arrindell & Ettema, 2005). Even though anxiety and depression subscales are highly correlated in the validation study of the SCL-90-R, the subscales have been shown to measure two separate dimensions.

#### Autism Spectrum Quotient

The Autism Spectrum Quotient (AQ; Baron-Cohen et al., 2001; Hoekstra et al., 2008) is a self-report questionnaire that measures autism traits and consists of 50 items scored on a 4-point Likert-type scale (1 = *definitely agree* to 4 = *definitely disagree*). Items are rescored to a 0 or 1 following the algorithm of the AQ, with 1 indicating autistic-like behavior, resulting in a total score ranging from 0 to 50. Psychometric properties of the AQ are satisfactory (Baron-Cohen et al., 2001; Hoekstra et al., 2008).

### Procedure

The present study is approved by the ethical commission of the University of Amsterdam. All participants gave written consent for participation in the study. Participants completed a series of questionnaires, including the CAT-Q-NL, the SCL-90-R, and the AQ. After 1.5 to 2 years, participants filled in the same questionnaires. Participants filled in the questionnaires online or using paper-and-pencil, according to their own preference, at home. Participants received a small monetary reward for participating in the study. Data at baseline have already been described in van der Putten et al. (2023b). The first measurement took place between September 2018 and October 2020. Almost all participants that were included in the present study filled in their questionnaire before March 2020. In March 2020, the first lockdown was started in the Netherlands due to the COVID-19 pandemic. The second measurement was conducted between November 2020 and February 2022.

### Community involvement

Four older and/or autistic adults were involved in the overarching “Autism & Aging” study (Geurts et al., 2021) to advise on, among other topics, recruitment of participants, study design, information letters, and interpretation of results. Researchers met the group about 4 times a year for 5 years and the group was compensated for their contributions. For this study, they gave their thoughts on our hypotheses, and shared their own experiences with respect to the hypothesized relationship between camouflaging and mental health difficulties and their interpretation of the findings.

### Statistical analyses

All analyses were preregistered at Aspredicted (#89077). We executed multilevel regression analyses in RStudio (RStudio Team, 2020) using the “lme4” Package (Bates et al., 2015). While we aimed to estimate random intercepts and slopes, only random intercepts could be estimated due to the number of observations. We controlled for biological sex, age, and AQ-score on T1 in all analyses. For the first research question, we included mental health difficulties (total/anxiety/depression) as dependent variable and time, camouflaging (total/assimilation) at T1, and Time × Camouflaging (total/assimilation) at T1 as predictors in the multilevel model. The effect of time represents the change in the outcome variable. The interaction between time and T1 predictor represents whether the change over time depends on the T1 predictor. For the second research question, we included camouflaging (total/assimilation) as dependent variable and time, mental health difficulties (total/anxiety/depression) at T1, and Time × Mental Health Difficulties (total/anxiety/depression) at T1 as predictors in the multilevel model. This resulted in six multilevel models for each research question. *P* values were Bonferroni-Holm corrected for these multiple comparisons (k = 6).

In addition to our preregistered analyses, in order to investigate the added value of the observed interaction effects, we tested whether there was more evidence for models without the interaction between Time × Predictor (H0) or with this interaction (H1). We compared these models using the Akaike information criterion (AIC), Bayesian information criterion (BIC), and marginal R-squared and by executing Bayesian analyses using the “bayestestR” package (Makowski et al., 2019). We interpreted the Bayes factors (BF) according to the guidelines described in van Doorn and colleagues (2021), that is, BF > 10 represents strong evidence for H1, BF > 3 moderate evidence for H1, and a BF between 3 and 0.33 is inconclusive, BF < 0.33 is moderate evidence for H0, and BF < 0.01 is strong evidence for H0. Thus, these additional analyses were solely added to be able to compare the two models, but not to test specific betas.

Finally, in our preregistration we indicated that, if possible, we would explore a total model with longitudinal structural equation models. However, given that we only had two-time measurements, there needed to be a substantial level of change between variables for these models to be informative. As there was only modest change between variables, we did not execute these analyses.

## Results

### Does camouflaging on T1 predict a change in mental health between T1 and T2?

**Figure 1.**
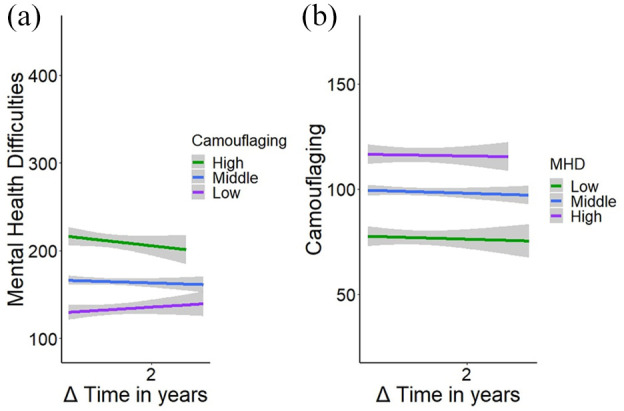
Visual representation of (a) the interaction between camouflaging as a predictor for a change in mental health difficulties and (b) the interaction between mental health difficulties (MHD) as a predictor for a change in camouflaging. For interpretive purposes, the predictor variable is transformed into a categorical variable with three levels mean ± 1 standard deviation. The length of the lines differs as it represents a time interval which varies between 1.3 and 3.3 years.

First, we investigated whether initial levels of camouflaging predicted a change in mental health difficulties over time. The results are visualized in Figure 1(a) and reported in Table 2 for mental health difficulties, anxiety, and depression as outcome variables and camouflaging and assimilation at baseline as predictor variables.

**Table 2.** Results of the multilevel models with mental health difficulties, depression, and anxiety as outcome variables, camouflaging or assimilation and time as predictor variables with significant results in bold.

Outcome: SCL-90-R total							
	Intercept	Time	CAT-Q-NL T1	AQ T1	Sex T1	Age T1	Time × CAT-Q-NL T1
β	**81.47**	10.29	**0.82**	**0.54**	5.18	–0.37	–**0.12**
CI	**43.91, 119.03**	2.28, 18.30	**0.62, 1.03**	**0.10, 0.97**	–4.71, 15.07	–0.78, 0.04	–**0.20,**–**0.04**
*p*	<0**.001**	**0.012**	<0**.001**	**0.016**	0.304	0.079	**0.003**
Adj. *p*	<0**.001**	0.072	<0**.001**	**0.048**	0.608	0.237	**0.018**
	Intercept	Time	Assimilation T1	AQ T1	Sex T1	Age T1	Time × Assimilation T1
β	**7.01**	1.15	**0.10**	0.06	1.49	–0.05	–0.01
CI	**1.70, 12.33**	–0.07, 2.38	**0.07, 0.13**	0.00, 0.13	0.10, 2.88	–0.11, 0.01	–0.03, –0.00
*p*	**0.01**	0.065	<0**.001**	**0.055**	**0.036**	0.081	**0.028**
Adj. *p*	**0.02**	0.325	<0**.001**	0.110	0.186	0.237	0.140
Outcome: SCL-90-R depression							
	Intercept	Time	CAT-Q-NL T1	AQ T1	Sex T1	Age T1	Time × CAT-Q-NL T1
β	**13.64**	1.84	**0.16**	0.11	2.73	–0.08	–0.02
CI	**4.17, 23.10**	–0.30, 3.98	**0.10, 0.21**	0.00, 0.23	0.25, 5.21	–0.18, 0.03	–0.04, –0.00
*p*	**0.005**	0.092	<0**.001**	**0.055**	**0.031**	0.148	**0.079**
Adj. *p*	**0.02**	0.368	<0**.001**	0.110	0.186	0.237	0.237
	Intercept	Time	Assimilation T1	AQ T1	Sex T1	Age T1	Time × Assimilation T1
β	**11.13**	1.31	**0.40**	**0.32**	2.40	–**0.13**	–0.04
CI	**1.97, 20.30**	–1.01, 3.63	**0.29, 0.51**	**0.22, 0.42**	–0.03, 4.84	–**0.23,** –**0.03**	–0.10, 0.03
*p*	**0.017**	0.267	<0**.001**	<0**.001**	0.053	0.012	0.256
Adj. *p*	**0.02**	0.534	<0**.001**	<0**.001**	0.204	**0.048**	0.512
Outcome: SCL-90-R anxiety							
	Intercept	Time	CAT-Q-NL T1	AQ T1	Sex T1	Age T1	Time × CAT-Q-NL T1
β	**7.01**	1.15	**0.10**	0.06	1.49	–0.05	–0.01
CI	**1.70, 12.33**	–0.07, 2.38	**0.07, 0.13**	0.00, 0.13	0.10, 2.88	–0.11, 0.01	–0.03, –0.00
*p*	**0.01**	0.065	<0**.001**	**0.055**	**0.036**	0.081	**0.028**
Adj. *p*	**0.02**	0.325	<0**.001**	0.110	0.186	0.237	0.140
	Intercept	Time	Assimilation T1	AQ T1	Sex T1	Age T1	Time × Assimilation T1
β	**7.35**	0.47	**0.21**	**0.17**	1.38	–**0.08**	–0.02
CI	**2.12, 12.59**	–0.87, 1.81	**0.14, 0.27**	**0.11, 0.23**	–0.01, 2.77	–**0.14,** –**0.03**	–0.06, 0.02
*p*	**0.006**	0.489	<0**.001**	<0**.001**	0.051	**0.004**	0.331
Adj. *p*	**0.02**	0.534	<0**.001**	<0**.001**	0.204	**0.020**	0.512

CI: confidence interval; adj. *p*: Bonferroni-Holm-adjusted *p* value; CAT-Q-NL: Dutch Camouflaging Autistic Traits Questionnaire; AQ: Autism Spectrum Quotient; Sex: Biological Sex. In bold are the predictors for which the adjusted *p* value was significant.

#### Camouflaging as a predictor for a change in mental health

The results show that camouflaging at baseline was significantly associated with mental health difficulties (β = 0.62), anxiety (β = 0.10), and depression (β = 0.16) at baseline (all adjusted *p*s < 0.001). This indicates that people with higher camouflaging scores start with more mental health difficulties, anxiety, and depression. While there is no significant effect of time on mental health difficulties, anxiety, and depression (adjusted *p*s > 0.06), there is a significant interaction between time and camouflaging (β = –0.12 adjusted *p*s < 0.05). The negative regression coefficient indicates that higher camouflaging scores at baseline predict a stronger decrease in mental health difficulties over time, while lower scores predict an increase in mental health difficulties. We reran this analysis to explore whether controlling for baseline level of mental health difficulties changed the pattern of findings (see Supplemental Table S2). In these exploratory analyses, the interaction between total camouflaging and time remained significant (β = –0.08, *p* = 0.026). However, it is important to note that this *p* value was not adjusted for multiple comparisons. This interaction between time and camouflaging was not found for anxiety or depression (adjusted *p*s > 0.140).

#### Assimilation as a predictor for a change in mental health

Assimilation at baseline was also significantly associated with mental health difficulties (β = 1.92), anxiety (β = 0.25), and depression (β = 0.41) at baseline (all adjusted *p*s < 0.001). This indicates that people with high assimilation scores start with more mental health difficulties, anxiety, and depression. The results show that there is no significant effect of time on mental health difficulties, anxiety, and depression (adjusted *p*s *=* 1.00) and no significant interaction between time and assimilation on mental health difficulties, anxiety, and depression (adjusted *p*s > 0.196). This indicates that level of assimilation does not seem to be a predictor for a change in mental health difficulties.

### Does mental health on T1 predict a change in camouflaging between T1 and T2?

Second, we investigated whether someone’s initial level of mental health difficulties predicted a change in camouflaging. The results are visualized in Figure 1(b) and described in Table 3 for camouflaging and assimilation as outcome variables, and with mental health difficulties, anxiety, and depression at baseline as predictor variables.

**Table 3.** Results of the multilevel models with camouflaging and assimilation as outcome variables and mental health difficulties, depression, anxiety and time as predictor variables.

Outcome: CAT-Q-NL total							
	Intercept	Time	SCL-90-R T1	AQ T1	Sex T1	Age T1	Time × SCL-90-R T1
β	**64.63**	0.84	**0.22**	0.11	3.27	–0.23	–0.01
CI	**47.82, 81.44**	–2.36, 4.04	**0.17, 0.27**	–0.07, 0.30	–1.38, 7.92	–0.42, –0.03	–0.03, 0.01
*p*	<0**.001**	0.608	<0**.001**	0.226	0.168	**0.020**	0.309
Adj. *p*	<0.001	1.00	<0**.001**	0.266	1.00	0.080	0.831
	Intercept	Time	Depression T1	AQ T1	Sex T1	Age T1	Time × Depression T1
β	**79.31**	–0.13	**0.70**	0.14	3.16	–0.25	–0.02
CI	**62.64, 95.97**	–2.74, 2.48	**0.50, 0.91**	–0.04, 0.32	–1.69, 8.02	–0.45, –0.06	–0.09, 0.06
*p*	<0**.001**	0.924	<0**.001**	0.133	0.201	0.012	0.611
Adj. *p*	<0**.001**	1.00	<0**.001**	0.266	1.00	0.072	0.831
	Intercept	Time	Anxiety T1	AQ T1	Sex T1	Age T1	Time × Anxiety T1
β	**77.83**	0.53	**1.31**	0.17	2.64	–0.24	–0.07
CI	**61.46, 94.19**	–1.99, 3.04	**0.95, 1.67**	–0.01, .35	–2.17, 7.46	–0.44, –0.05	–0.20, 0.06
*p*	**<0.001**	0.682	<0**.001**	0.066	0.282	**0.015**	0.277
Adj. *p*	<0**.001**	1.00	<0**.001**	.198	1.00	0.075	0.831
Outcome: CAT-Q-NL assimilation							
	Intercept	Time	SCL-90-R T1	AQ T1	Sex T1	Age T1	Time × SCL-90-R T1
β	**8.62**	1.55	**0.08**	**0.40**	–0.12	–0.00	**–0.01**
CI	**3.75, 13.48**	–0.02, 3.312	**0.06, 0.09**	**0.32, 0.47**	–1.10, 1.41	–0.05, 0.05	–0.02, –0.00
*p*	**0.001**	**0.053**	<0**.001**	<0**.001**	0.813	0.894	**0.006**
Adj. *p*	**0.002**	0.32	<0**.001**	<0**.001**	1.00	1.00	**0.036**
	Intercept	Time	Depression T1	AQ T1	Sex T1	Age T1	Time × Depression T1
β	**9.24**	1.09	**0.26**	0.53	–0.13	–0.01	–0.05
CI	**4.80, 13.68**	–0.31, 2.50	**0.20, 0.32**	0.46, 0.60	–1.30, 1.05	–0.06, 0.03	–0.09, –0.01
*p*	<0**.001**	0.128	<0**.001**	<0.001	0.833	0.595	0.012
Adj. *p*	<0**.001**	0.64	<0**.001**	<0.001	1.00	1.00	0.05
	Intercept	Time	Anxiety T1	AQ T1	Sex T1	Age T1	Time × Anxiety T1
β	**10.35**	1.07	**0.38**	**0.55**	–0.11	–0.02	–0.09
CI	**5.85, 14.85**	–0.31, 2.44	**0.27, 0.49**	**0.48, 0.62**	–1.31, 1.10	–0.07, 0.03	–0.16, –0.02
*p*	<0**.001**	0.128	<0**.001**	<0**.001**	0.862	0.514	0.010
Adj. *p*	<0**.001**	0.64	<0**.001**	<0**.001**	1.00	1.00	0.05

CI: Confidence interval; adj. *p*: Bonferroni-Holm-adjusted *p* value; SCL-90-R: Symptom Checklist-90 Revised; AQ: Autism Spectrum Quotient; Sex: Biological Sex. In bold are the predictors for which the adjusted *p* value was significant.

#### Mental health as a predictor for a change in camouflaging

The results show that mental health difficulties (β = 0.22), anxiety (β = 0.86) and depression (β = 0.54) at baseline were significantly associated with camouflaging at baseline (all adjusted *p*s < 0.001). This indicates that people with more mental health difficulties start with higher levels of camouflaging. However, there is no significant effect of time on camouflaging (adjusted *p* = 1.00) and no significant interaction between time and mental health difficulties, anxiety, and depression (adjusted *p* > 0.95). This indicates that someone’s level of mental health difficulties, anxiety, or depression does not predict a change in camouflaging.

#### Mental health as a predictor for a change in assimilation

The results show that mental health difficulties (β = 0.07), anxiety (β = 0.41), and depression (β = 0.25) at baseline were significantly associated with assimilation at baseline (all adjusted *p* < 0.001). This indicates that people with more mental health difficulties start with a higher level of assimilation. While there is no significant effect of time on assimilation (adjusted *p* > 0.24), there was a significant interaction between time and mental health difficulties (β = –0.01, adjusted *p* = 0.036). This indicates that people with more initial mental health difficulties show a stronger decrease in assimilation strategies while people with less mental health difficulties show an increase in assimilation. However, when we reran the analysis and controlled for baseline level of assimilation strategies, there was no significant interaction effect between time and mental health difficulties (see Supplemental Table S2). For anxiety and depression, there was no significant interaction with time (adjusted *p* > 0.30).

##### Comparing models with and without interaction between time and predictor

To explore the extent of the observed significant interaction effects, we compared whether there is more evidence for the models without interaction between time and predictor variables (H0) or for the models with an interaction between time and predictor variables (H1). All fit indices resulting from these analyses are summarized in Table 4. The Bayes Factors (BFs) are also shown in Supplemental Figure S1.

**Table 4.** Fit indices for the comparison between multilevel models without the interaction between time and predictor and with this interaction.

Model		Outcome	Predictor	AIC	BIC	*R* ^2^	Δ*R*^2^	BF
**1**	**W/o interaction**	**SCL-90-R**	**CAT-Q-NL**	**6752.62**	**6788.60**	**0.185**	**-**	**-**
	**W interaction**	**SCL-90-R**	**CAT-Q-NL**	**6750.25**	**6790.74**	**0.192**	**0.007**	**3.51**
2	W/o interaction	SCL-90-R	Assimilation	6730.32	6766.31	0.226	-	-
	W interaction	SCL-90-R	Assimilation	6730.76	6771.24	0.228	0.002	0.28
3	W/o interaction	Depression	CAT-Q-NL	4971.00	5006.99	0.140	-	-
	W interaction	Depression	CAT-Q-NL	4977.10	5017.59	0.143	0.003	0.19
4	W/o interaction	Depression	Assimilation	4949.73	4985.71	0.182	-	-
	W interaction	Depression	Assimilation	4955.40	4995.88	0.183	0.001	0.08
5	W/o interaction	Anxiety	CAT-Q-NL	4226.42	4262.41	0.156	-	-
	W interaction	Anxiety	CAT-Q-NL	4231.87	4272.35	0.160	0.004	0.45
6	W/o interaction	Anxiety	Assimilation	4218.00	4253.98	0.170	-	-
	W interaction	Anxiety	Assimilation	4225.12	4265.60	0.170	0.000	0.06
7	W/o interaction	CAT-Q-NL	SCL-90-R	5793.71	5829.70	0.231	-	-
	W interaction	CAT-Q-NL	SCL-90-R	5802.22	5842.70	0.233	0.002	0.07
8	W/o interaction	CAT-Q-NL	Depression	5820.23	5856.22	0.169	-	-
	W interaction	CAT-Q-NL	Depression	5826.67	5867.16	0.170	0.001	.04
9	W/o interaction	CAT-Q-NL	Anxiety	5814.60	5850.59	0.180	-	-
	W interaction	CAT-Q-NL	Anxiety	5819.03	5859.52	0.182	0.002	0.07
**10**	**W/o interaction**	**Assimilation**	**SCL-90-R**	**4477.40**	**4513.38**	**0.326**	**-**	**-**
	**W interaction**	**Assimilation**	**SCL-90-R**	**4482.14**	**4522.63**	**0.387**	**0.051**	**1.19**
11	W/o interaction	Assimilation	Depression	4494.65	4530.64	0.375	-	-
	W interaction	Assimilation	Depression	4496.67	4537.15	0.417	0.042	0.93
12	W/o interaction	Assimilation	Anxiety	4518.27	4554.26	0.349	-	-
	W interaction	Assimilation	Anxiety	4518.73	4559.21	0.388	0.039	1.12

AIC: Akaike information criterion; BIC: Bayesian information criterion; *R*^2^: marginal *R*-squared; BF: Bayes Factor; CAT-Q-NL: Dutch Camouflaging Autistic Traits Questionnaire; SCL-90-R total: Symptom Checklist-90 Revised; w: model with interaction; w/o: model with-out interaction. Please note that the models with a statistical significant interaction coefficient are displayed in bold, as these analyses were specifically executed to investigate the added value of this interaction.

#### Camouflaging as predictor for a change in mental health difficulties

For Model 1, with mental health difficulties as outcome variable and camouflaging as predictor variable, the results show that there is moderate evidence for the model with an interaction opposed to the model without interaction. However, all fit indices (AIC, BIC, and Δ*R*^2^) showed that the model without an interaction was preferred. This implies that the additional explained variance from the interaction was small. For Model 5, with anxiety as outcome variable and camouflaging as predictor, the Bayesian results were inconclusive but all fit indices were in favor of the model without interaction. Therefore, this points toward evidence that the model without interaction is preferred for the change in anxiety. For Models 2, 3, 4, and 6 (that is, the models with assimilation strategies as a predictor, and one model with depression as outcome variable), there was moderate evidence for the model without interaction. Based on all fit indices (AIC, BIC and Δ*R*^2^), the model without interaction is preferred.

#### Mental health difficulties as predictor for a change in camouflaging

For Models 7, 8 and 9, with camouflaging as outcome variable, there was moderate evidence for the model without interaction over the model with interaction based on the Bayesian analyses and these results were in line with the fit indices (AIC, BIC, and Δ*R*^2^). For Models 10, 11, and 12, with assimilation as outcome variable, the BFs were inconclusive and the Δ*R*^2^ indicated that the model with interaction explained more variance compared with the model without interaction (Δ*R*^2^ between 0.039 and 0.051). However, based on the AIC and BIC, the model without interaction is preferred. Therefore, for the change in assimilation strategies, it remains unclear whether the model with or without interaction is preferred, while for the change in camouflaging the model without interaction seems most suitable.

##### Exploratory analyses: the masking and compensation subscale

In addition to our preregistered analyses, we analyzed our research questions also for the masking and compensation subscale. The results of these analyses are shown in Supplemental Tables S3A, S3B, and S4. As these analyses were exploratory, we did not control for multiple comparisons. The results show that there were no meaningful effects of initial level of masking and compensation on the change in mental health difficulties. That is, only a significant interaction was found between time and initial level of masking on total mental health difficulties. However, model comparisons showed that the model without interaction was preferred over the model with interaction. Furthermore, there was no effect of initial mental health difficulties on the change in masking or compensation.

## Discussion

In this study, we investigated the direction of the association between camouflaging and mental health difficulties using a longitudinal study design. All quantitative knowledge about this association thus far was based on cross-sectional studies (Cook et al., 2021). Therefore, we tested whether (1) camouflaging was a predictor for a change in mental health difficulties and (2) mental health difficulties were a predictor for a change in camouflaging. Our finding indicated that initial levels of mental health difficulties did not seem to predict a change in camouflaging, and additionally, camouflaging itself seemed to remain stable over time. However, initial level of camouflaging was a predictor for a change in mental health difficulties. Unexpectedly, for people with higher initial levels of camouflaging, there was a decrease in mental health difficulties, while for people with lower initial camouflaging there was an increase in mental health difficulties. While these findings could imply that depending on one’s initial level of camouflaging there may be some beneficial effects of camouflaging, the effects were small, and within this study design the conclusions one can draw regarding directionality are still limited. The findings, nonetheless, do show that one needs to be cautious with drawing strong conclusions about direct effects of camouflaging on mental health, either positive or negative.

We found that at baseline camouflaging was associated with baseline mental health difficulties, which is in line with previous cross-sectional studies (Cook et al., 2021). However, when we investigated the change in mental health difficulties over time in the longitudinal data, the results could indicate that camouflaging might be related to a change in mental health over time, but the direction was opposite to what we initially expected, and the effects were rather small. This unexpected finding might be best explained using the words of a member of our autistic think tank who reflected on our findings in writing:^3^

More than 10 years ago, I was diagnosed with autism and after a few years of open communication I became silent. . . . The price [of camouflaging] is high, the process complicated. So, every day I have to weigh what brings me more, and yet in the end, this balancing act gives me enough. . . . As long as our society is not yet able to act maturely enough to embrace neurodiversity, I will have to fix this myself.

Thus, even though camouflaging feels exhausting and can have a negative impact on someone’s self-image (Libsack et al., 2021), it may also be a necessary strategy to cope with the demands of our non-autistic society. To what extent autistic people are accepted by others can play an important role in the mental health difficulties someone experiences (Cage et al., 2018). By camouflaging more, people may experience less direct negative reactions from their non-autistic peers, which could in turn contribute to fewer mental health difficulties.

Next to considering whether camouflaging predicted a change in mental health difficulties, we investigated an alternative hypothesis, namely, if initial levels of mental health difficulties predicted a change in camouflaging behavior. Our results showed that whether camouflaging changed over time was not associated with someone’s initial mental health difficulties. These findings differed from what would be expected based on interviews with autistic teenagers (Chapman et al., 2022), in which teenagers reported that their mental health difficulties caused them to camouflage. However, in this study, we included participants who were all (older) autistic adults between 30 and 90 years of age, instead of teenagers (see, for an example, Lundin Remnélius & Bölte, 2024). Therefore, the development of these camouflaging strategies could have occurred earlier in life, and we may have not been able to capture this directionality in our data. Another qualitative study showed that mainly the sophistication of the strategies changed over time (Loo et al., 2023). However, with the CAT-Q-NL we measure which camouflaging strategies one reports to use and specifically how often certain camouflaging strategies are used, but not how sophisticated the execution is. Therefore, changes in camouflaging may have not been captured with this specific questionnaire. Thus, when interpreting the results, it is important to take into account this characteristic of the measurement instrument. Note that while the construct validity of the CAT-Q should be further studied, the consensus across the literature is that the CAT-Q mainly measures camouflaging intent even though a few items in the CAT-Q focus specifically on intent and the discrepancy mainly measures camouflaging “success” (Cook et al., 2021; Williams, 2022). Including more facets of camouflaging such as the number of contexts someone camouflages in, the effort it requires, or whether one experiences camouflaging to be voluntary or necessary could provide more useful insights.

In addition, it is important that our results about changes in assimilation strategies were inconsistent, as the frequentist analyses, model comparison fit indices, and Bayesian results were not in line. That is, based on frequentist analyses mental health difficulties seemed to be significantly associated with a change in assimilation; however, the Bayes factors were inconclusive and model fit indices indicated a preference for the model without interaction above the model with interaction. This makes it difficult to interpret these findings. In addition, as items of the assimilation subscales have been found to be closely associated with social anxiety (McKinnon et al., 2024), it could also be difficult to disentangle these concepts. Whether changes in assimilation strategies may or may not be affected by mental health difficulties should be studied further. Also, when we conducted exploratory analyses with the compensation and masking subscales, we found similar results. While this could imply that these subscales do not change differently over time, confirmative studies should be conducted to gain insight into the longitudinal differences across these subscales. Moreover, overall, we did not observe large changes in mental health difficulties or camouflaging behaviors over time. Executing a comparable study with groups in which more change occurs, for example, in a mental health care setting, may result in more noticeable changes over time, making effects of the predictor variables more apparent. Furthermore, the fact that our sample consisted of people of 30 years or older could explain that we only found an association between camouflaging and mental health difficulties at T1, but no direct effect over time. That is, forming this relation may have taken place earlier in life, for example, during adolescence, while later in life, camouflaging strategies are reported to have become more sophisticated and automatic processes (Loo et al., 2023). Conducting a study during earlier developmental stages, such as adolescence, one might gain more insight into the temporal relationship. Also, by widening the age range, one may gain insight in the potentially non-linear relationship between camouflaging and mental health over time.

**Figure 2.**
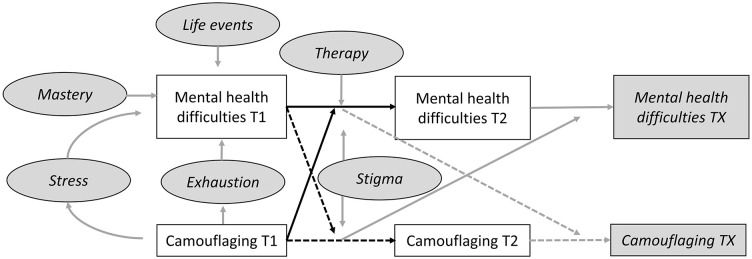
The white boxes and black arrows the associations are shown between camouflaging and mental health as investigated in the current study and in gray boxes, ovals, and arrows the potential ways that different variables may interact with these associations. The relationships depicted in gray arrows were not tested in this study, but are hypothesized to be of importance in this association based on previous research. A selection of potential variables has been made, but more or other variables can also play a role.

With the current study design, we could investigate the association between camouflaging and mental health difficulties over time. This is an important step toward establishing directionality and causality (Lervåg, 2019). However, next to temporal precedence (cause occurs before the effect) and that cause and effect need to be associated, it needs to be determined whether there are alternative explanations. There is a wide variety of factors that can impact the association between camouflaging and mental health, as we visualized in Figure 2. The associations that were tested in this study are shown in white boxes and in gray ovals and boxes, the potential additional associations are shown that could be investigated to draw further conclusions about directionality and causality. That is, other factors can influence someone’s level of or the change in mental health difficulties, for example (negative) life events, feelings of mastery, or receiving therapy (Fuld, 2018; van Heijst et al., 2020). In addition, previous research suggested that stigma, stress, and exhaustion may play an important role in how camouflaging and mental health are related (Hull et al., 2017; Livingston et al., 2019; Perry et al., 2022). Therefore, camouflaging may not directly impact mental health, but camouflaging may contribute to decreased mental health through factors such as stigma, exhaustion, or stress. In addition, other factors could be considered as outcome variables, such as fatigue or burnout symptoms. Exploring a range of factors (as mentioned in Figure 2) that might impact the relationship between camouflaging and mental health can help both the clinician and the client in creating a holistic perspective of how camouflaging and mental health interact for a specific client.

Finally, in our cross-sectional study, we recently showed that the association between camouflaging and mental health differs in strength, with some subgroups of autistic adults showing stronger associations between mental health difficulties and camouflaging than others (van der Putten et al., 2023b). Investigating whether changes over time vary between subgroups and thus whether camouflaging may have a positive effect for some, while it has a negative effect for others could be another important research question to answer. Focusing on various psychosocial variables which have been shown to be useful in predicting camouflaging scores (Zhuang et al., 2024), such as stigma, negative life events or the strength of one’s autistic identity, would be recommended to disentangle for whom camouflaging is associated with mental health difficulties. By gaining insight into the coping strategies or emotion regulation used by certain subgroups, one can better understand and support people for whom camouflaging is most strongly associated with mental health difficulties.

### Strengths and limitations

This study has several important strengths. First, due to our longitudinal study design we could make a first step in investigating directionality in the association between camouflaging and mental health. Second, our study was conducted in a large group of autistic adults with a wide age range with an equal representation of both males and females, with only around 5% of participants dropping out between the first and second measurement. Third, our analysis plan was preregistered and included sophisticated analyses for estimating changes over time while incorporating the varying time intervals. Also, we used additional Bayesian analyses to explore how much evidence there is for the alternative hypothesis as opposed to the null hypothesis. Finally, autistic adults were involved in the study to help with interpretation of these findings.

However, there are also some important caveats to consider. As aforementioned, conclusions about causality are limited, as we still cannot confirm that the decrease in mental health difficulties is attributed solely to someone’s level of camouflaging with two data points. Due to the association between camouflaging and mental health (Cook et al., 2021), people who reported using a lot of camouflaging strategies, in general, also started with a higher level of mental health difficulties. For them, there may be more room to improve their mental health and/or show fewer extreme responses at the second measurement (regression to mean). Without receiving mental health care, people with many mental health difficulties tend to report less difficulties on a second measurement and vice versa (Arrindell & Ettema, 2005). Additional sensitivity analyses showed that when we controlled for baseline level of the outcome variables, the effect of the predictors decreased, which indicates that the decrease in mental health difficulties could be explained by the initial level of the outcome variable (regression to the mean). To establish whether a change in camouflaging is followed by a change in mental health difficulties, we require data collected over more than two timepoints (see Figure 2). While the use of two time measurements is an important first step to provide insights into changes over time at the group level (Parsons & McCormick, 2024), we would recommend including three (or more) points over time as a next step for future studies (Collins et al., 1998; Hamaker et al., 2015). Including more, but also longer, time intervals would improve the robustness of conclusions we can draw about directionality. As this was the first study investigating the relationship between camouflaging and mental health over time, our findings need to be replicated to strengthen the robustness of our conclusions.

Furthermore, our study sample consists of adults of 30 to 90 years old, who are highly educated, mostly white, mainly identify with binary genders and have been diagnosed with autism mostly at a later age. More research is necessary to find out whether these findings generalize to other groups, which include, for example, other stigmatized minorities, based on sexuality, gender, or ethnicity. In addition, replicating these findings in teenagers, younger adults, or adults who are diagnosed in childhood would be informative for the generalizability of these findings. Finally, since data collection of the second measurement took place during the COVID-19 pandemic, this may have influenced the mental health difficulties experienced by autistic adults as well as their use of camouflaging behaviors, for example a decreased necessity to adjust facial expressions due to wearing face masks or reduced opportunities for direct social contact which was replaced by virtual contact. It has been shown that the COVID-19 pandemic may have had varying effects on mental health of autistic adults (Scheeren et al., 2023).

In summary, our findings indicated that level of mental health difficulties did not seem to be associated with a change in camouflaging, while higher levels of camouflaging seemed to be associated with a decrease in mental health. However, this effect was small. Gathering more measurements over time, including potential third variables, and investigating this association in mental health care settings are important next steps to take toward gaining insight into the mechanisms underlying the association between camouflaging and mental health. Until there are stronger group-based conclusions about this association, it is important that clinicians explore together with their autistic clients what the potential negative but also positive consequences of camouflaging are, as experienced by this specific person, together with the reasons for doing so. This could help with creating a holistic perspective of how camouflaging and mental health interact for this person.

## Supplemental Material

sj-docx-1-aut-10.1177_13623613251347104 – Supplemental material for Camouflaging in autism: A cause or a consequence of mental health difficulties? Supplemental material, sj-docx-1-aut-10.1177_13623613251347104 for Camouflaging in autism: A cause or a consequence of mental health difficulties? by Wikke J van der Putten, Audrey JJ Mol, Tulsi A Radhoe, Carolien Torenvliet, Joost A Agelink van Rentergem, Annabeth P Groenman and Hilde M Geurts in Autism

## Notes

1. There are differences in preferences between identity-first (autistic person) or person-first (person with autism) language, which can also differ between countries (Buijsman et al., 2022; Bury et al., 2023; Kenny et al., 2016). We used identity-first language throughout the article.

2. Different terms are used to describe camouflaging, such as masking, compensation, and passing as non-autistic (PAN). For clarity, we used the term camouflaging in this article.

3. For the original quote, see Supplementary materials.
