# Supplementary material for: Camouflaging in autism: A cause or a consequence of mental health difficulties?
Source: Autism. 2025 Jun 28;29(10):2604–17. doi: 10.1177/13623613251347104 (PMC12417600; doi:10.1177/13623613251347104)
Supplement: sj-docx-1-aut-10.1177_13623613251347104 – Supplemental material for Camouflaging in autism: A cause or a consequence of mental health difficulties? [file sj-docx-1-aut-10.1177_13623613251347104.docx]

**Supplementary materials**

Camouflaging in autism: A cause or a consequence of mental health difficulties?

Table S1. Characteristics and test statistics between the sample that was included in the study and the sample that dropped-out between the first and second measurement.

|  | **Included sample** | **Drop-out** | **Test statistics** |
| --- | --- | --- | --- |
| Biological sex (m/f/o) T1 | 174/157/1 | 9/12/1 | χ² (2) = 1.75, *p* =.42 |
|  | M (SD; range) | M (SD; range) |  |
| Age T1 | 52.4 (12.4; 30-84) | 52.3 (13.8; 31-78) | *t*(23.31) =.05, *p* = .96 |
| AQ T1 | 34.7 (7.4; 10-48) | 30.8 (8.4; 16-45) | *t*(23.17) = 2.06, *p* = .05 |
| CAT-Q-NL T1 | 98.6 (26; 29-169) | 94.5 (29.8; 42-161) | *t*(23.15) = .62, *p* = .54 |
| SCL-90-R T1 | 169.6 (51.1; 93-397) | 173.4 (56.9; 106-320) | *t*(23.27) =- .31, *p* =.76 |

m/f/o: male/female/other, AQ: Autism Spectrum Quotient, CAT-Q-NL: Dutch Camouflaging Autistic Traits Questionnaire, SCL-90-R: Symptom Checklist-90 Revised.

*Table S2.* Results of the sensitivity analyses in which we controlled for baseline level of the outcome variable.

| **Outcome: SCL-90-R total** | | | | | | | | |
| --- | --- | --- | --- | --- | --- | --- | --- | --- |
|  | Intercept | **SCL-90-R T1** | Time | CAT-Q-NL T1 | AQ T1 | Sex T1 | Age T1 | **Time***  **CAT-Q-NL T1** |
| *β* | 11.49 | **.92** | 6.43 | .05 | .11 | .04 | .11 | **-.08** |
| CI | -4.81; 27.78 | **.88; .96** | -.82; 13.69 | -.06; .15 | -.18; .40 | -3.70; 3.77 | -.29; .02 | **-.16; -.01** |
| *p* | .167 | **<.001** | .082 | .373 | .455 | .985 | .09 | **.026** |
| **Outcome: CAT-Q-NL Assimilation** | | | | | | | | |
|  | Intercept | **Assimilation T1** | Time | **SCL-90-R T1** | **AQ T1** | Sex T1 | Age T1 | Time*  SCL-90-R T1 |
| *β* | -.01 | **.63** | -1.09 | **.02** | **.34** | -.54 | -.03 | .00 |
| CI | -3.77; 3.75 | **.58; .69** | -2.23; .06 | **.01; .03** | **.29; .40** | -1.50; .42 | -.07; .01 | -.00; .01 |
| *p* | .997 | **<.001** | .063 | **.002** | **<.001** | .272 | .168 | .189 |

CI: Confidence interval, p: p-value, SCL-90-R: Symptom Checklist-90 Revised, AQ: Autism Spectrum Quotient, Sex: Biological Sex. In bold are the predictors for which the p-value was significant.

*Table S3A.* Results of the multilevel models with mental health difficulties, depression and anxiety as outcome variables and compensation or masking as predictor variables with significant results in bold.

| **Outcome: SCL-90-R total** | | | | | | | |
| --- | --- | --- | --- | --- | --- | --- | --- |
|  | Intercept | Time | Compensation T1 | AQ T1 | Sex T1 | Age T1 | Time*  Compensation T1 |
| *β* | **104.50** | 2.63 | **1.41** | **1.11** | 4.83 | **-.48** | -.14 |
| CI | **66.46, 142.55** | -5.83, 11.09 | **1.02, 1.81** | **.74, .148** | -5.48, 15.14 | **-.90, -.06** | -.38, .11 |
| *p* | **<.001** | .542 | **<.001** | **<.001** | .358 | **.026** | .274 |
|  | Intercept | Time | Masking T1 | AQ T1 | Sex T1 | Age T1 | Time*  Masking T1 |
| *β* | **128.58** | 5.43 | **1.32** | 0.29 | 7.33 | -.42 | **-.21** |
| CI | **90.22, 166.94** | -1.52, 12.38 | **0.80, 1.84** | -0.06, 0.63 | -3.20, 18.87 | -0.86, .01 | **-.41, -.01** |
| *p* | **<.001** | .126 | **<.001** | .100 | .172 | .058 | **.044** |
| **Outcome: SCL-90-R depression** | | | | | | | |
|  | Intercept | Time | Compensation T1 | AQ T1 | Sex T1 | Age T1 | Time*  Compensation T1 |
| *β* | **19.62** | -.06 | **.24** | .20 | **2.72** | -.10 | -.00 |
| CI | **10.11, 29.13** | -2.31, 2.20 | **.14, .35** | .11, .30 | **.15, 5.29** | -.21, .00 | -.07, .06 |
| *p* | **<.001** | .962 | **<.001** | <.001 | **.038** | .057 | .977 |
|  | Intercept | Time | Masking T1 | AQ T1 | Sex T1 | Age T1 | Time*  Masking T1 |
| *β* | **23.13** | .90 | .25 | .05 | **3.17** | -.09 | -.03 |
| CI | **13.63, 32.63** | -.94, 2.75 | .12, .38 | -.04, .14 | **.58, 5.76** | -.19. .02 | -.08, .03 |
| *p* | **<.001** | .337 | <.001 | .273 | **.016** | .115 | .330 |
| **Outcome: SCL-90-R anxiety** | | | | | | | |
|  | Intercept | Time | Compensation T1 | AQ T1 | Sex T1 | Age T1 | Time*  Compensation T1 |
| *β* | 9.11 | .11 | .17 | .12 | 1.45 | -.06 | -.01 |
| CI | 4.69, 15.30 | -1.18, 1.40 | .11,.23 | .07, .17 | .02, 2.88 | -.12, -.01 | -.05, .03 |
| *p* | <.001 | .868 | <.001 | <.001 | .047 | .029 | .572 |
|  | Intercept | Time | Masking T1 | AQ T1 | Sex T1 | Age T1 | Time*  Masking T1 |
| *β* | 12.24 | .45 | .17 | .02 | **1.73** | -.05 | -.02 |
| CI | 6.92, 17.56 | -.61, 1.51 | .10, .25 | -.03, .07 | **.28, 3.17** | -.11, .01 | -.05, .01 |
| *p* | <.001 | .402 | <.001 | .410 | **.019** | .074 | .224 |

CI: Confidence interval, adj. p: Bonferroni-Holm adjusted p-value, CAT-Q-NL: Dutch Camouflaging Autistic Traits Questionnaire, AQ: Autism Spectrum Quotient, Sex: Biological Sex. In bold are the predictors for which the p-value was significant.

*Table S3B.* Results of the multilevel models with compensation and masking as outcome variables and mental health difficulties, depression and anxiety as predictor variables.

| **Outcome: CAT-Q-NL Compensation** | | | | | | | |
| --- | --- | --- | --- | --- | --- | --- | --- |
|  | Intercept | Time | SCL-90-R T1 | AQ T1 | Sex T1 | Age T1 | Time*  SCL-90-R T1 |
| *β* | **22.87** | -.25 | **.07** | -.00 | 1.32 | **-.11** | .00 |
| CI | **15.16, 30.59** | -1.70, 1.19 | **.05, .10** | -.07, .06 | -.83, 3.48 | **-.20, -.02** | -01, .01 |
| *p* | **<.001** | .730 | **<.001** | .942 | .228 | **.014** | .624 |
|  | Intercept | Time | Depression T1 | AQ T1 | Sex T1 | Age T1 | Time* Depression T1 |
| *β* | **28.80** | -.25 | **.21** | .01 | 1.38 | **-.12** | .01 |
| CI | **21.24, 36.37** | -1.43, .92 | **.11, .30** | -.06, .07 | -.85, 3.61 | **-.21, -.03** | -.02, .04 |
| *p* | **<.001** | .671 | **<.001** | .848 | .226 | **.008** | .520 |
|  | Intercept | Time | Anxiety  T1 | AQ T1 | Sex T1 | Age T1 | Time*  Anxiety T1 |
| *β* | **27.49** | -.15 | **.45** | .00 | 1.08 | **-.11** | .01 |
| CI | **20.08, 34.90** | -1.30, 1.00 | **.29, .61** | -.06, .07 | -1.12, 3.28 | **-.20, -.03** | -.04, .07 |
| *p* | **<.001** | .798 | **<.001** | .883 | .337 | **.012** | .646 |
| **Outcome: CAT-Q-NL Masking** | | | | | | | |
|  | Intercept | Time | SCL-90-R T1 | AQ T1 | Sex T1 | Age T1 | Time*  SCL-90-R T1 |
| *β* | **27.96** | .34 | **.06** | -.05 | 1.53 | **-.12** | -.00 |
| CI | **20.58, 35.33** | -.98, 1.67 | **.04, .08** | -.11, .01 | -.53, 3.60 | **-.21, -.04** | -.01, .00 |
| *p* | **<.001** | .613 | **<.001** | .120 | .145 | **.004** | .193 |
|  | Intercept | Time | Depression T1 | AQ T1 | Sex T1 | Age T1 | Time* Depression T1 |
| *β* | **32.01** | -.05 | **.18** | -.04 | 1.52 | **-.13** | -.01 |
| CI | **24.89, 39.13** | -1.13, 1.03 | **.09, .27** | -.10, .02 | -.58, 3.62 | **-.22, -.05** | -.04, .02 |
| *p* | **<.001** | .924 | **<.001** | .177 | .156 | **.003** | .401 |
|  | Intercept | Time | Anxiety  T1 | AQ T1 | Sex T1 | Age T1 | Time*  Anxiety T1 |
| *β* | 30.97 | .22 | .39 | -.01 | 1.31 | **-.13** | -.04 |
| CI | 23.96, 37.98 | -.83, 1.27 | .24, .54 | -.10, .02 | -.78, 3.40 | **-.21, -.04** | -.09, .01 |
| *p* | **<.001** | .683 | **<.001** | .175 | .219 | **.004** | .155 |

CI: Confidence interval, adj. p: Bonferroni-Holm adjusted p-value, SCL-90-R: Symptom Checklist-90 Revised, AQ: Autism Spectrum Quotient, Sex: Biological Sex. In bold are the predictors for which the p-value was significant.

*Table S4.* Fit indices for the comparison between multilevel models without the interaction between time and predictor and with this interaction.

| **Model** | | **Outcome** | **Predictor** | **AIC** | **BIC** | **R^2^** | **ΔR^2^** | **BF** |
| --- | --- | --- | --- | --- | --- | --- | --- | --- |
| 1 | W/o interaction | SCL-90-R | Compensation | 6764.06 | 6800.04 | .154 | - | - |
|  | W interaction | SCL-90-R | Compensation | 6767.18 | 6807.67 | .154 | .000 | .071 |
| 2 | W/o interaction | SCL-90-R | Masking | 6790.63 | 6826.62 | .095 | - | - |
|  | W interaction | SCL-90-R | Masking | 6791.31 | 6831.80 | .098 | .003 | .300 |
| 3 | W/o interaction | Depression | Compensation | 4983.42 | 5019.41 | .109 | - | - |
|  | W interaction | Depression | Compensation | 4990.39 | 5030.87 | .109 | .000 | .039 |
| 4 | W/o interaction | Depression | Masking | 4995.38 | 5031.37 | .080 | - | - |
|  | W interaction | Depression | Masking | 5001.80 | 5042.29 | .081 | .001 | .063 |
| 5 | W/o interaction | Anxiety | Compensation | 4234.24 | 4270.22 | .132 | - | - |
|  | W interaction | Anxiety | Compensation | 4241.99 | 4282.48 | .132 | .000 | .046 |
| 6 | W/o interaction | Anxiety | Masking | 4249.32 | 4285.31 | .101 | - | - |
|  | W interaction | Anxiety | Masking | 4256.33 | 4296.81 | .103 | .002 | .082 |
| 7 | W/o interaction | Compensation | SCL-90-R | 4766.52 | 4802.51 | .159 | - | - |
|  | W interaction | Compensation | SCL-90-R | 4777.42 | 4817.91 | .158 | .001 | .044 |
| 8 | W/o interaction | Compensation | Depression | 4788.41 | 4824.40 | .103 | - | - |
|  | W interaction | Compensation | Depression | 4796.31 | 4836.80 | .103 | .000 | .048 |
| 9 | W/o interaction | Compensation | Anxiety | 4775.87 | 4811.86 | .130 | - | - |
|  | W interaction | Compensation | Anxiety | 4782.87 | 4823.35 | .129 | .001 | .043 |
| 10 | W/o interaction | Masking | SCL-90-R | 4682.88 | 4718.87 | .113 | - | - |
|  | W interaction | Masking | SCL-90-R | 4692.50 | 4732.99 | .114 | .001 | .091 |
| 11 | W/o interaction | Masking | Depression | 4691.04 | 4727.03 | .090 | - | - |
|  | W interaction | Masking | Depression | 4698.83 | 4739.32 | .090 | .000 | .056 |
| 12 | W/o interaction | Masking | Anxiety | 4683.55 | 4719.54 | .104 | - | - |
|  | W interaction | Masking | Anxiety | 4688.91 | 4729.39 | .105 | .001 | .108 |

AIC: Akaike information criterion, BIC: Bayesian information criterion. R^2^: marginal R-squared, BF: Bayes Factor, CAT-Q-NL: Dutch Camouflaging Autistic Traits Questionnaire, SCL-90-R total: Symptom Checklist-90 Revised, w: model with interaction, w/o: model with-out interaction.


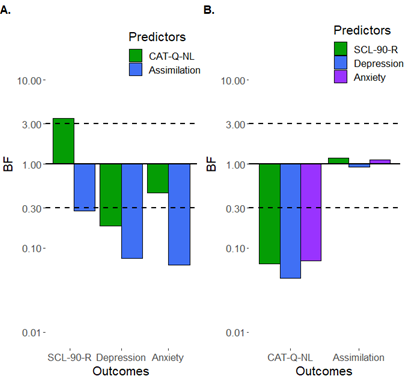


*Figure S1.* Bayes Factors (BFs) for the comparison between the multilevel models without the interaction between time and predictor (H0) and with this interaction (H1). Note that BFs < .30 indicates moderate evidence for H0 (i.e., no interaction) and BFs > 3 indicates moderate evidence for H1 (i.e., with interaction), BFs between .30 and 3 indicates inconclusive evidence.

**Original Dutch quote from discussion:**

Ruim 10 jaar geleden kreeg ik de diagnose ASS en na een aantal jaren open communiceren deed ik er het zwijgen toe. (…) Dus de prijs is hoog, het traject ingewikkeld.

Elke dag is dus weer een afweging wat me meer oplevert, en toch levert deze balanceer act me uiteindelijk genoeg op. (…) Zolang deze samenleving zich niet volwassener kan gedragen ten aanzien van neurodiversiteit, moet ik het zelf maar oplossen.
